# Responses of stance leg muscles induced by support surface translation during gait

**DOI:** 10.1016/j.heliyon.2022.e10470

**Published:** 2022-08-30

**Authors:** Shiho Fukuda, Hitoshi Oda, Taku Kawasaki, Yasushi Sawaguchi, Masakazu Matsuoka, Ryo Tsujinaka, Koichi Hiraoka

**Affiliations:** a Graduate School of Comprehensive Rehabilitation, Osaka Prefecture University, Habikino City, Osaka, Japan; b College of Health and Human Sciences, Osaka Prefecture University, Habikino City, Osaka, Japan

**Keywords:** Gait, Perturbation, Electromyographic response, Cognition, Postural control

## Abstract

This study determined the presence of the muscle responses to the support surface translation in the stance leg during gait and examined the effect of the direction and time point of the translation and that of the cognitive process on the responses. The rectus femoris (RF), biceps femoris (BF), soleus (SOL), and tibialis anterior (TA) muscles in the stance leg were tested. There was no significant effect of cognitive process on the electromyographic (EMG) activity induced by the translation of the support surface. In all muscles except the SOL, the EMG amplitude increased 0–300 ​ms after the support surface translation at the initial stance (IS) or middle stance (MS) of the tested leg. This means that the EMG activity in the leg muscles other than the SOL occurs after the support surface translation at the IS or MS no matter the direction of the translation. The EMG amplitude was not changed after the translation at the late stance, indicating that the translation does not influence the EMG amplitude at the double limb support phase with the tested leg behind the other. In the SOL, the EMG amplitude increased after the backward translation at the IS and after the forward translation at the MS, but decreased after the forward translation at the IS, indicating that the support surface translation-induced change in the EMG amplitude of the SOL is dependent on its direction. The change in the EMG amplitude of the TA and RF induced by the forward translation was greatest when the translation was given at the IS. In the SOL, the decrease in the EMG amplitude after the forward translation and the increase in the amplitude after the backward translation were greatest at the IS. Taken together, the change in the EMG amplitude induced by the support surface translation is greatest when the translation is given at the IS. The increase in the EMG amplitude in the TA and RF after the forward translation was greater than that after the backward translation at the IS, indicating that the EMG activity of the frontal leg muscles after the forward translation is greater when the translation is given at the IS.

## 1 Introduction

Postural responses to the perturbation are crucial to maintain the upright posture during gait. The responses have been induced by the acceleration or deceleration of the treadmill (Dietz et al., 1984; Berger et al., 1984; Kagawa et al., 2011), movement of the slip roller (Marigold and Patla, 2002), or horizontal support surface translation (Nashner, 1980; Tang et al., 1998; Tang and Woollacott, 1999; Ferber et al., 2002) in previous studies. In those previous studies, forward translation of the support surface, the deceleration of the support surface of the treadmill, or the rolling of the slip roller causing forward slip of the foot has been given to induce the backward leaning of the body (backward perturbation). Opposite direction of the perturbation has been given to induce the forward leaning of the body (forward perturbation).

The responses of the leg muscles were induced by the perturbation at the initial stance (IS) or midstance (MS), but those were absent when the perturbation was given at the late (LS) or terminal stance (Nashner, 1980; Dietz et al., 1984; Berger et al., 1984; Tang et al., 1998; Tang and Woollacott, 1999; Ferber et al., 2002; Kagawa et al., 2011). The response of the tibialis anterior (TA) muscle was induced by the backward perturbation at the IS (Nashner, 1980; Dietz et al., 1984; Berger et al., 1984; Tang et al., 1998; Tang and Woollacott, 1999; Ferber et al., 2002; Kagawa et al., 2011), but was absent after the forward perturbation (Nashner, 1980; Dietz et al., 1984; Berger et al., 1984). The response of the TA was present after the backward perturbation, but that was absent after the forward perturbation at the MS (Nashner, 1980). The activity level of the gastrocnemius (GC) or soleus (SOL) muscle was decreased by the backward perturbation at the IS or MS (Nashner, 1980; Dietz et al., 1984; Berger et al., 1984; Tang et al., 1998; Tang and Woollacott, 1999; Ferber et al., 2002; Kagawa et al., 2011). The response of the GC was induced by the forward perturbation at the IS or MS (Nashner, 1980; Dietz et al., 1984; Berger et al., 1984). There are several previous findings that the response of the quadriceps or biceps femoris (BF) muscle was induced by the backward perturbation, but no findings on the response to the forward perturbation have been reported (Tang et al., 1998; Tang and Woollacott, 1999; Ferber et al., 2002; Kagawa et al., 2011).

According to those previous findings, the presence and absence of the stance leg muscle responses to the perturbation during gait is phase- and direction-dependent. However, in most studies, the responses were identified via visual inspection (Nashner, 1980; Dietz et al., 1984; Berger et al., 1984). In some studies, the responses were identified through standard deviation (SD) method; the time bins of the rectified electromyographic (EMG) trace, in which the EMG amplitude exceeded mean +1 SD of the average EMG amplitude of the unperturbed trials in the corresponding time window, were considered to be the time in which the EMG response was present (Tang et al., 1998; Tang and Woollacott, 1999; Ferber et al., 2002; Kagawa et al., 2011).

However, the EMG responses were not detected in some participants via SD method (Tang and Woollacott, 1999), causing that statistical test to determine the presence of the EMG response across all participants is impossible. The determination of the onset and offset of the EMG response via mathematical algorithm has been proposed, but this methodology is not for determining of the presence and absence of the response (Rashid et al., 2019). Moreover, there is no study that statistically tested the effect of the direction and time point of the perturbation on the magnitude of those responses. In the present study, presence and absence of the EMG response to the support surface translation, and the effect of the direction and time point of the translation on the responses of the upper and lower leg muscles in the stance leg during gait were determined via statistical test across the participants.

The long-latency response of the SOL to the support surface translation in stance is mediated by the transcortical pathways (Taube et al., 2006). The postural response to the support surface translation is prepared in advance according to a previous finding that the cortical response to the translation was modulated by time prediction (Jacobs and Horak, 2007; Jacobs et al., 2008). The response of the leg muscles to the support surface translation is prepared by central set (Horak and Nashner, 1986; Horak et al., 1989). The prediction of time point and direction of the support surface translation reduced the late component of the ankle EMG response to the backward translation of the support surface (Matsuoka et al., 2020). Cognitive load (silent backward counting in steps of seven) reduced the body sway induced by the vibration over the gastrocnemius muscles causing twitch of those muscles (Andersson et al., 2002), and reduced the late components of the ankle muscle responses to the perturbation in stance (Rankin et al., 2000). Those findings indicate that cognitive process contributes to the postural response in stance. Cognitive process may even influence the preparation process of the response to the postural perturbation during gait.

Several studies applied cognitive load on human gait. The participants repeatedly subtracted the number from assigned number during gait (Abbud et al., 2009; Al-Yahya et al., 2009; Soangra and Lockhart, 2017). In spite of those previous findings, there is no studies that tested the influence of cognitive load on the muscle responses to the perturbation during gait. Thus, we tested our hypothesis that the muscle response to the perturbation is prepared by the central set during gait through examining the effect of cognitive load on the response.

## 2 Methods

### 2.1 Participants

Participants were 14 healthy males aged 34.0 ± 7.9 years. There are gender differences in physical characteristics (Hamill et al., 1977) and motor performance (Thomas and French, 1985). Thus, to exclude variability in motor performance caused by gender difference, only males were recruited. All participants had no history of neurological or musculoskeletal diseases. Written informed consent was obtained from all participants. The experiment was conducted according to Declaration of Helsinki and was approved by the ethics committee of Osaka Prefecture University (approval number: 2020-122).

### 2.2 Walkway

**Figure 1:**
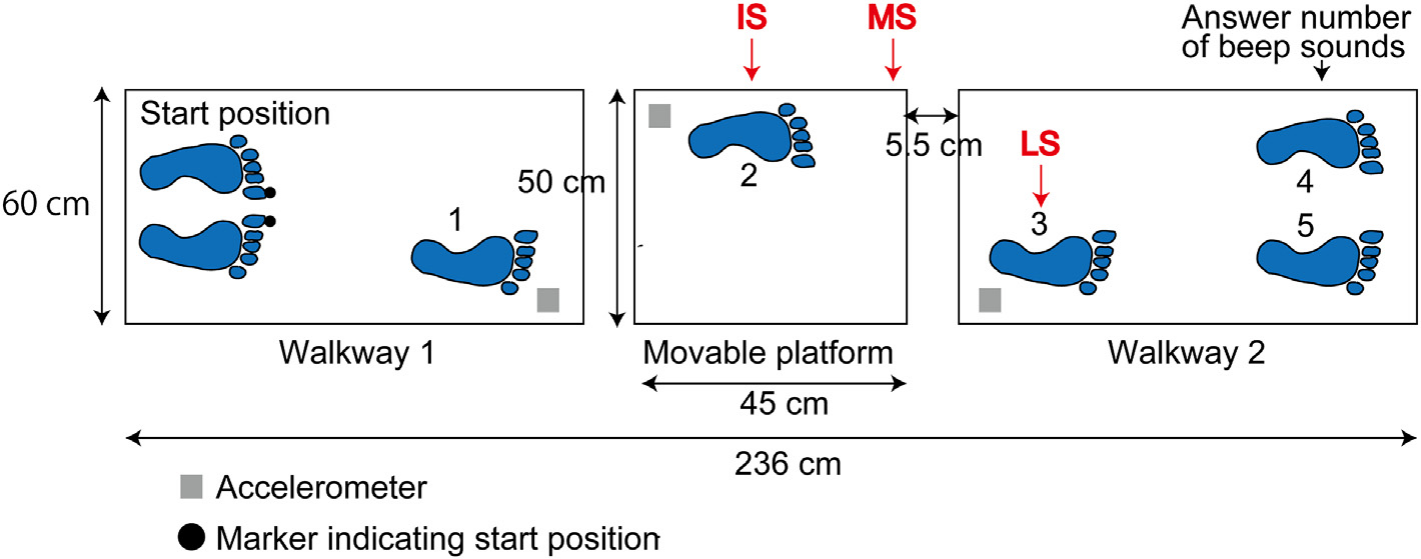
The experimental setup. The numbers indicate the order of the steps during the gait task. IS; support surface translation and beep sounds are given at the left heel contact over the movable platform (second step), MS; translation and sounds are given at the half time between the left (second step) and second right heel contact (third step). LS; translation and sounds are given at the second right heel contact over the walkway 2 (third step).

A movable platform with 50 cm width and 45 cm length was placed on the ground. The support surface of this movable platform translated 5 cm in the forward or backward direction. The duration of the translation was 88 ​ms in the forward direction and 85 ​ms in the backward direction. The translation of the support surface reached a peak velocity of 122 cm/s at the moment 40 ​ms after the onset of the support surface translation in the forward direction. The translation reached a peak velocity of 121 cm/s at the moment 42 ​ms after the onset of the support surface translation in the backward direction. Walkways were placed in front of (walkway 1) and beyond (walkway 2) the platform (Figure 1). The size of each walkway was 60 cm width and 90 cm length. There was a space with 5.5 cm length between the end of each walkway and that of the platform. Taken together, the total length of the walkway, including the platform surface, was 236 cm.

### 2.3 Auditory pulses

Earphones were inserted into the participants' ears. Beep sound pulses with sound-pressure level of 40 dB and frequency of 1 kHz were generated by these earphones simultaneously with the support surface translation. The number of the beep sound pulses was 5, 6, 7, or 8. The interval between the pulses was 80 ​ms.

### 2.4 Measurements

The surface electrodes recording the EMG activity were placed over the bellies of the left RF, BF, SOL, and TA. The distance between the electrodes was 2 cm. The signals from those electrodes were amplified via amplifiers with band-pass filter between 50 Hz and 1 kHz (MEG-2100; Nihon Kohden, Tokyo, Japan). A foot switch was attached over the tip of each big toe to detect the toe off (TO). An accelerometer was placed on the walkway 1 to detect the first right heel contact (HC). Another accelerometer was placed on the support surface of the platform to detect the left HC over the support surface of the platform. The other accelerometer was placed on the walkway 2 to detect the second right HC. The analogue signals from those sensors were digitized at a sampling rate of 1 kHz (PowerLab/8sp and 2sp; ADInstruments, Colorado Spring, CO, USA). The digitized signals were stored on a personal computer.

### 2.5 Gait without translation session

Both the gait without translation session and experimental session were conducted in a same day. The gait without translation session was conducted before the experimental session. The participants walked from the start position through to the end of the walkway 10 times (Figure 1). An experimenter asked the participants to walk through to the end of the walkway with the velocity slightly faster than the comfortable velocity. This velocity was applied for the experiment, because the response to the perturbation was great when the participants walked at this velocity in our preliminary experiments. The start position of the big toes was marked with markers at the beginning of the walkway so that they initiated gait from the same position every trial. After starting the gait, the right heel firstly contacted the walkway 1, the left heel contacted the support surface of the platform next, the right heel contacted over the walkway 2, and then, final two steps were made to reach at the end of the walkway (Figure 1). The trials, in which the participants initiated gait with the left leg, were discarded and those unsuccessful trials were retried after the completion of the scheduled 10 trials.

### 2.6 Experimental session

The participants walked from the start position through to the end of the walkway in the experimental session (Figure 1). The support surface of the platform translated forward or backward in the translation condition. The support surface translation was triggered at one of three time points. The translation was triggered when the left heel contacted the platform; translation at the IS. The platform translation was triggered when the right heel contacted over the walkway 2; translation at the LS. At the moment, at which the translation was given at the IS or LS, the gait was in the double-limb support phase. The platform translation was triggered at the half time between the IS and LS; translation at the MS. At this time, the gait was in the left single support phase. The time at the MS was calculated using the data in the gait without translation session. Taken together, 6 tasks (two translation directions with three translation time points) were conducted. Ten trials were conducted in each time and direction of the support surface translation (translation condition). In addition, 10 trials without the translation were conducted (non-translation condition). Thus, 70 trials were conducted in each cognitive load condition.

Beep sound with 5, 6, 7 or 8 pulses were given at the time the support surface translation onset. In the cognitive load condition, the participants verbally answered the number of the pulses that they recognized when the translation was given after the end of the gait. In this condition, cognitive load must have been imposed because they had to count and memorize the number of the beep sound pulses. In the non-cognitive load condition, they did not answer the number of the pulses. Before beginning each trial, an experimenter informed the participants whether they had to answer the number or not in the next trial. Seventy trials of the cognitive load condition and seventy trials of the non-cognitive load condition were conducted. Totally, 140 trials were conducted. The translation direction, time, and cognitive load were assigned each trial with a random order.

### 2.7 Identification of EMG response

The EMG traces were rectified and the rectified ten EMG traces in each task were averaged. The EMG trace, whose amplitude exceeded mean + 1SD or 3SD of the amplitude of the unperturbed trials in the corresponding time window, was considered to be the EMG response (Tang et al., 1998; Tang and Woollacott, 1999; Brauer et al., 2002; Fujimoto et al., 2013; Little and Woollacott, 2015). In the present study, the rectified and averaged EMG trace in the trials without support surface translation was subtracted from the EMG trace in the trials with support surface translation (subtracted EMG trace) to identify the EMG response to the support surface translation in each task. The average amplitude of the subtracted EMG trace in the time window 0–100 ​ms before the translation onset was considered to be the background EMG (BEMG) amplitude. The EMG amplitude after the translation was averaged each 5 ​ms bin. Three or more consecutive time bins (15 ​ms or more) of the subtracted EMG trace, whose amplitude exceeded + 3SD of BEMG amplitude, were considered to be the time window in which the EMG amplitude increased. Three or more consecutive time bins of the subtracted EMG trace, whose amplitude was below mean - 3SD of the BEMG amplitude, were considered to be the time window in which the EMG amplitude decreased. The number of the participants, in which the increase or the decrease in the EMG amplitude after the translation was present according to 3D method, was counted. Then, appearing probability of the increase and the decrease in the EMG across the participants was calculated.

### 2.8 Change in EMG activity after translation

Appearing probability of the EMG response to the support surface translation during gait, expressed as the number of the trials with the response divided by the total number of the trials, was much smaller than 1.0 in most translation conditions (Tang and Woollacott, 1999). This indicates that the response is not present for all participants. To conduct the statistical analysis of the EMG amplitude, the data must be present in all the participants. Thus, in the present study, the difference between the BEMG amplitude and the EMG amplitude after the translation was statistically determined for the data across all participants. The mean amplitude of the EMG 0–300 ​ms after the translation onset was calculated. Then, the difference between the mean EMG amplitude 0–300 ​ms after the support surface translation and the BEMG amplitude was tested by paired t-tests to identify the increase and decrease in the EMG amplitude immediately after the translation. The alpha level was set at 0.05.

### 2.9 Effect of time, direction, and cognition

The effect of the translation time point (IS, MS, and LS), translation direction (forward and backward translation), and cognitive load (cognitive load and non-cognitive load) on the EMG amplitude 0–300 ​ms after the translation was tested by three-way repeated measures of ANOVA. The result of Greenhouse–Geisser’s correction was reported whenever Mauchly’s test of sphericity was significant. When there was a significant interaction between the main effects, test of simple main effect was conducted. If the test of the simple main effect revealed a significant main effect, multiple comparison test (Bonferroni’s test) followed it.

## 3 Results

### 3.1 Appearing probability of responses

**Figure 2:**
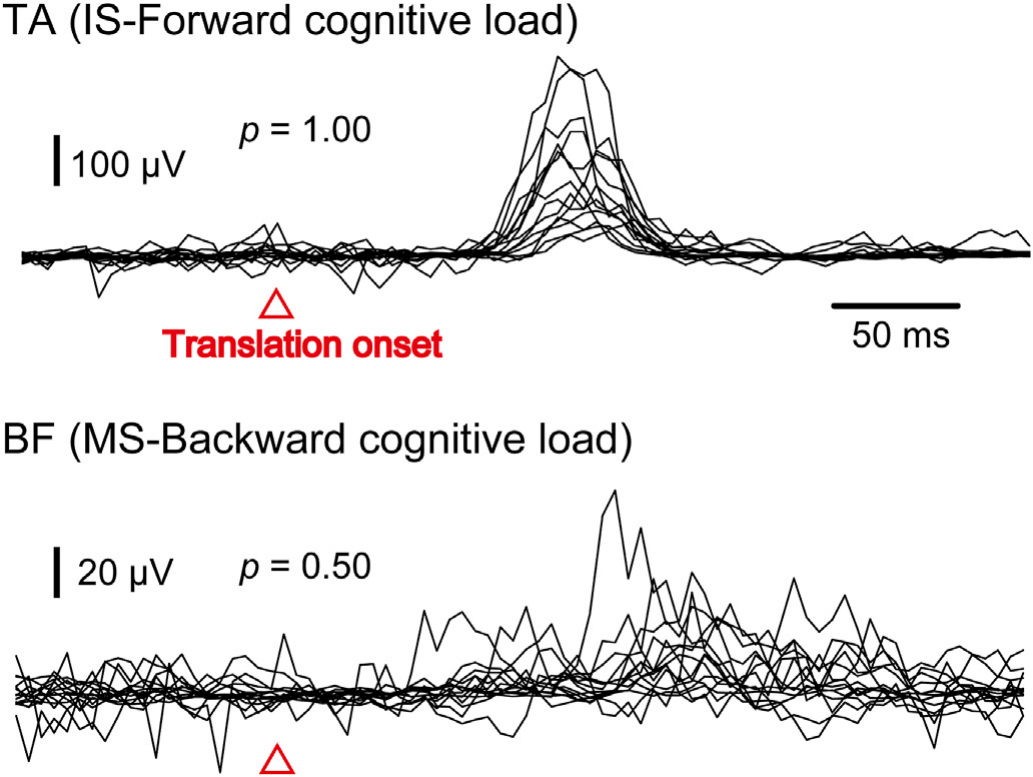
Examples of the subtracted EMG traces in the time window between 100 ​ms before and 300 ​ms after the translation across all participants. Each trace indicates a trace in each participant. The upper traces indicate the subtracted EMG traces in the TA before and after the forward translation at the IS in the cognitive load condition. An obvious response is present across the participants. Appearing probability of the EMG response is 1.00 in those traces. The lower traces indicate the subtracted EMG traces in the BF before and after the backward translation at the MS in the cognitive load condition. The responses are variable among the participants. Appearing probability of the EMG response is 0.50 in those traces.

Examples of the subtracted EMG traces in the time window between before and after the translation across all participants are shown in Figure 2. For the EMG traces in the TA before and after the forward translation at the IS in the cognitive load condition, an obvious response was present across the participants. Appearing probability of the EMG response was 1.00 in those traces. For the EMG traces in the BF before and after the backward translation at the MS in the cognitive load condition, the responses were variable among the participants. Appearing probability of the EMG response was 0.50 in those traces.

The appearing probability of the responses determined by the SD method is shown in Table 1. The probability varied across the muscles across the tasks. The excitatory response was present across all the participants for the forward translation at the IS in both cognitive load conditions and at the MS in the non-cognitive load condition in the TA. The excitatory response was present across all the participants for the backward translation at the IS in the non-cognitive load condition in the SOL. In the other conditions, there were certain number of the participants in which the responses were absent.

**Table 1:** Probability of EMG response appearance.

	Non-cognitive load						Cognitive load					
	Backward			Forward			Backward			Forward		
	IS	MS	LS	IS	MS	LS	IS	MS	LS	IS	MS	LS
Excitation												
TA	0.29	0.86	0.43	1.00	1.00	0.43	0.14	0.71	0.43	1.00	0.93	0.50
SOL	1.00	0.43	0.00	0.79	0.71	0.07	0.93	0.36	0.00	0.64	0.71	0.00
BF	0.57	0.86	0.21	0.79	0.71	0.21	0.36	0.50	0.36	0.71	0.79	0.29
RF	0.86	0.79	0.36	0.93	0.64	0.43	0.57	0.86	0.36	0.93	0.64	0.43
Inhibition												
TA	0.00	0.36	0.21	0.00	0.43	0.21	0.00	0.36	0.21	0.00	0.57	0.00
SOL	0.36	0.14	0.00	0.86	0.29	0.07	0.36	0.00	0.00	0.93	0.43	0.00
BF	0.00	0.14	0.29	0.00	0.21	0.14	0.00	0.14	0.43	0.00	0.29	0.21
RF	0.21	0.07	0.21	0.36	0.21	0.29	0.14	0.07	0.21	0.36	0.14	0.29

TA, tibialis anterior; SOL, soleus; BF, biceps femoris; RF, rectus femoris; IS, translation at initial stance; MS, translation at midstance; LS, translation at late stance; Backward, backward translation; Forward, forward translation.

### 3.2 EMG amplitude 0–300 ms after translation

The mean BEMG amplitude and the mean EMG amplitude 0–300 ​ms after the perturbation are shown in Table 2. In all muscles except the SOL, the EMG amplitude 0–300 ​ms after the translation was significantly greater than the BEMG amplitude in either the IS or MS in either direction (p < 0.05). In the SOL, the EMG amplitude 0–300 ​ms after the translation was significantly greater than the BEMG amplitude for the backward translation at the IS, and for the forward translation at the MS (p < 0.05). The amplitude in the SOL was significantly less than the BEMG amplitude for the forward translation at the IS (p < 0.05). The effect of the translation was not significant when the translation was given at the LS across all muscles.

**Table 2:** EMG amplitude 0–300 ​ms after translation.

	TA (μV)							SOL (μV)							BF (μV)							RF (μV)						
	BEMG			EMG				BEMG			EMG				BEMG			EMG				BEMG			EMG			
Backward translation																												
IS	-1.2	±	5.0	3.0	±	2.0	∗	0.7	±	3.8	13.5	±	4.8	∗	-0.1	±	2.8	6.5	±	3.5	∗	-0.6	±	2.3	3.4	±	2.3	∗
MS	-0.1	±	3.3	5.9	±	7.0	∗	-0.8	±	7.0	1.9	±	3.9		-1.4	±	4.3	5.0	±	4.0	∗	0.0	±	2.5	2.4	±	2.0	∗
LS	0.9	±	3.4	1.3	±	3.1		2.6	±	6.3	0.9	±	2.1		-0.5	±	2.2	-0.9	±	3.5		-0.5	±	2.6	0.6	±	2.6	
Forward translation																												
IS	1.5	±	8.3	28.0	±	19.9	∗	0.4	±	2.1	-8.1	±	6.3	∗	0.8	±	3.6	5.5	±	3.2	∗	0.4	±	1.9	8.5	±	7.6	∗
MS	-0.5	±	3.9	8.7	±	7.9	∗	-0.7	±	2.9	3.4	±	4.6	∗	0.3	±	2.1	5.0	±	4.2	∗	-0.5	±	1.3	1.4	±	2.9	∗
LS	0.7	±	4.7	-0.1	±	3.4		2.3	±	5.8	0.9	±	2.2		-0.3	±	3.8	0.3	±	3.5		0.3	±	2.2	0.7	±	2.0	

Mean ± SD; ∗p < 0.05 (BEMG vs. EMG); BEMG; EMG amplitude 0–100 ms before translation; EMG; EMG amplitude 0–300 ms after translation; TA, tibialis anterior; SOL, soleus; BF, biceps femoris; RF, rectus femoris; IS, translation at initial stance; MS, translation at midstance; LS, translation at late stance.

### 3.3 Cognitive load and translation direction and time

**Figure 3:**
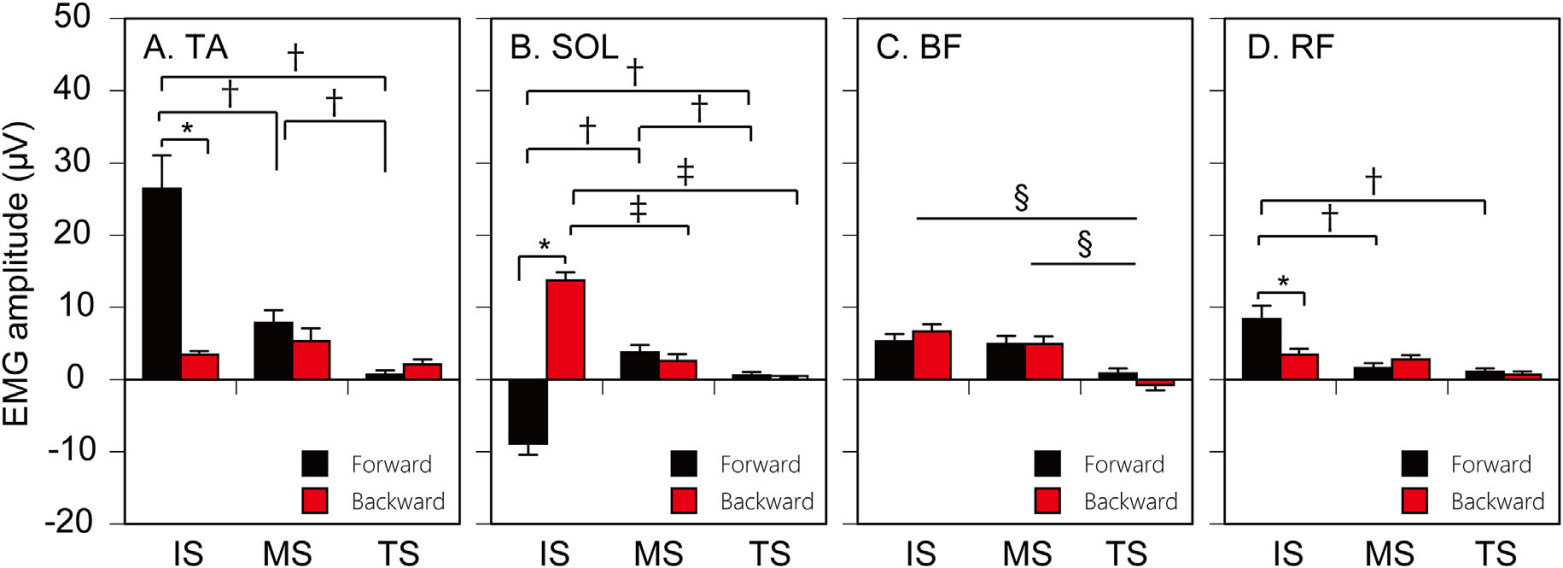
Effect of the translation direction and time on EMG amplitude. Because the main effect of cognitive load is insignificant, means of two cognitive load conditions are averaged. Bars indicate mean and error bars indicate standard error of mean. Asterisks indicate a significant difference between the forward and backward directions (p < 0.05). Daggers indicate a significant difference between the translation time points for the forward translation (p < 0.05). Double daggers indicate a significant difference between the translation time points for the backward translation (p < 0.05). Section marks indicate a significant difference between the translation time points conducted by multiple comparison test for the significant main effect of the time (p < 0.05).

The mean EMG amplitude of the TA in the time window 0–300 ​ms after the translation is shown in Figure 3A. There was a significant interaction between the effect of the direction and time [F(1.190, 15.464) = 23.125, p < 0.001] (Greenhouse–Geisser correction; Mauchly’s Test, p = 0.001). The test of the simple main effect revealed that the effect of the forward translation was greater than that of the backward translation at the IS [F(1,39) = 72.615, p < 0.001]. There was a significant main effect of the time for the forward translation [F(2,52) = 49.715, p < 0.001]. Multiple comparison test revealed that the EMG amplitude after the forward translation at the IS was significantly greater than that at the MS or LS (p < 0.001), and that at the MS was significantly greater than that at the LS (p = 0.001). There was no significant main effect of the cognition [F(1,13) = 0.290, p = 0.599].

The mean EMG amplitude of the SOL in the time window 0–300 ​ms after the translation is shown in Figure 3B. There was a significant interaction between the main effect of the direction and time [F(1.303, 16.941) = 90.169, p < 0.001] (Greenhouse–Geisser correction; Mauchly’s Test, p < 0.001). Test of simple main effect revealed that the EMG amplitude after the forward translation was significantly smaller than that after the backward translation at the IS [F(1, 33) = 332.058, p < 0.001]. There was a significant main effect of the translation time point both for the forward [F(2,51) = 37.245, p < 0.001] and backward directions [F(2,51) = 43.840, p < 0.001]. Multiple comparison test revealed that the EMG amplitude after the forward translation at the IS was significantly smaller than that at the MS or LS (p < 0.001), and that at the MS was significantly greater than that at the LS (p = 0.013). Another multiple comparison test revealed that the EMG amplitude induced by the backward translation at the IS was significantly greater than that at the MS or LS (p < 0.001). There was no significant main effect of the cognition [F(1,13) = 0.052, p = 0.823].

The mean EMG amplitude of the BF in the time window 0–300 ​ms after the translation is shown in Figure 3C. There was a significant main effect of the translation time point [F (2,26) = 17.029, p < 0.001]. The EMG amplitude at IS and MS was significantly greater than that at the LS (p < 0.001). There was no significant main effect of the cognition [F(1,13) = 0.086, p = 0.774].

The mean EMG amplitude of the RF in the time window 0–300 ​ms after the translation is shown in Figure 3D. There was a significant interaction between the effect of the direction and time [F(2,26) = 13.727, p < 0.001]. The test of the simple main effect revealed that the EMG amplitude after the forward translation was greater than that after the backward translation at the IS [F(1,32) = 21.590, p < 0.001]. There was a significant main effect of the time for the forward translation [F(2,41) = 21.157, p < 0.001]. Multiple comparison test revealed that the EMG amplitude after the forward translation at the IS was significantly greater than that after the MS and LS (p < 0.001). There was no significant main effect of the cognition [F(1,13) = 0.587, p = 0.457].

## 4 Discussion

In the present study, the presence of the EMG responses to the support surface translation during gait was determined, and the effect of the time point and direction and that of cognitive load on the EMG activity after the translation was examined. The responses to the support surface translation were present in the IS and MS but not in the LS. The change in the EMG activity of the stance leg muscles was greatest when the translation was given at the IS. The change in the EMG activity of the frontal leg muscles was dependent on the direction of the support surface translation particularly at the IS.

### 4.1 EMG responses

On the one hand, the response of the TA was induced by the backward perturbation at the IS (Nashner, 1980; Dietz et al., 1984; Berger et al., 1984; Tang et al., 1998; Tang and Woollacott, 1999; Ferber et al., 2002; Kagawa et al., 2011). On the other hand, the response of the GC was present, but that of the TA was absent after the forward perturbation at the IS (Nashner, 1980; Dietz et al., 1984; Berger et al., 1984). Those previous findings indicate that the presence or absence of the responses in the lower leg muscles is dependent on the perturbation direction.

However, in those previous studies, there are some methodological shortages. In some previous studies, the EMG responses were identified through visual inspection (Nashner, 1980; Dietz et al., 1984; Berger et al., 1984). In the other previous studies, the responses were identified through searching the EMG components whose amplitude was 1 SD above the average EMG amplitude of the unperturbed trials in the corresponding time window (SD method) (Tang et al., 1998; Tang and Woollacott, 1999; Ferber et al., 2002; Kagawa et al., 2011). The SD method identifies only the large responses, and thus, small responses in some participants may have not been detected. Indeed, it has been reported that the response was not observed in all participants (Tang and Woollacott, 1999). As consistent with this previous finding, the responses identified by the SD method were not present across all participants in the present study. Thus, the direction-dependent responses observed in those previous studies must not represent the responses across all participants.

To fix these methodological shortages, the presence of the change in the EMG amplitude induced by the perturbation was determined by testing the difference between the across-participants mean amplitude of the subtracted EMG traces before the translation (BEMG) and that immediately after the translation. The EMG amplitude 0–300 ms after the translation was greater than the BEMG amplitude at the IS and MS for all muscles other than the SOL. This finding indicates that the presence of the response is not direction-dependent (except the SOL) as inconsistent with previous findings (Nashner, 1980; Dietz et al., 1984; Berger et al., 1984; Tang et al., 1998; Tang and Woollacott, 1999; Ferber et al., 2002; Kagawa et al., 2011).

The reason for the conflicting finding between the previous studies using the SD method and the present study using statistical procedure across the participants is that the statistical procedure testing the data across the participants conducted in the present study sensitively detected the change in the EMG amplitude after the perturbation comparing with the identification of the EMG response using the SD method. The SD method only detected the prominent response. Thus, large responses must have been detected but small responses were not, causing direction-dependent identification of the responses in the previous studies. In contrast, in the present study, the difference between mean EMG amplitude in the time window 0–300 ​ms before the translation and that in the time window 0–300 ​ms after that across the participants was statistically tested to determine the presence and absence of the EMG response. This procedure revealed even small responses that were not detected by the SD method, causing absence of the direction-dependency of the presence of the changes in the EMG amplitude. Taken together, the present finding is first to show that the presence of the EMG activity after the perturbation in the leg muscles other than the SOL is not dependent on the direction of the perturbation.

### 4.2 Decrease in SOL activity

The EMG amplitude particularly in the SOL was decreased after the forward translation (backward perturbation) at the IS. This finding was consistent with previous findings that the EMG response in the SOL or GC was suppressed by the backward perturbation (Nashner, 1980; Dietz et al., 1984; Berger et al., 1984; Tang et al., 1998; Tang and Woollacott, 1999; Ferber et al., 2002; Kagawa et al., 2011). Accordingly, the SOL is the specific muscle that responds to the support surface translation differently from the other stance leg muscles. Suppression of the SOL motoneuron pool during the postural tasks has been reported; e.g., suppression of the H-reflex in the SOL before gait initiation (Hiraoka et al., 2005, 2006). Accordingly, the present finding may reflect a fact that the excitability of the SOL motoneuron pool is suppressed during the postural task.

In the TA, the EMG activity induced by the forward translation at the IS was greater than that induced by the backward translation at the same time, and the activity induced by the forward translation at the IS was greatest among the translation time points. This means that the TA responded strongly to the forward translation at the IS. The reciprocal inhibition of the SOL is induced by the stimulation of the common peroneal nerve (Crone et al., 1987, El-Tohamy and Sedgwick, 1983). The reciprocal inhibition is also induced by the antagonist contraction; the voluntary contraction of the TA suppressed the H-reflex in the SOL (Kagamihara and Tanaka, 1985). Accordingly, the decrease in the EMG amplitude in the SOL specifically after the forward translation at the IS may be explained by reciprocal inhibition produced by the great contraction of the antagonist (TA).

Nevertheless, this view must be handled with caution. The reciprocal inhibition from the SOL or GC to the TA was fourfold stronger than that from the TA to the GC or SOL (Yavuz et al., 2018). Based on this finding, the reciprocal inhibition of the TA induced by the contraction of the SOL must have been greater than that of the SOL induced by the contraction of the TA. In the present study, the increase in the EMG amplitude of the SOL induced by the backward translation did not induce reciprocal decrease of the EMG amplitude in the TA. Thus, the present finding is not in line with reciprocal inhibition occurring between the agonist and antagonist of the ankle during voluntary contraction.

### 4.3 Direction dependency

The effect of the startle response on the EMG response to the forward translation of the support surface was different from that to the backward translation (Nonnekes et al., 2013). The startle response accompanies the decrease in the reaction time of the voluntary motor response, indicating that the startle response is largely related to the motor process. Moreover, the process underlying the postural response and that underlying the startle response shares common neural circuits (Brown et al., 1991). Accordingly, the central process mediating the postural response to the backward translation is different from the process mediating the response to the forward translation in stance. In the present study, the direction-dependent EMG activity was present particularly when the translation was given at the IS; i.e., TA and RF responded more strongly to the forward translation, but the SOL more strongly responded to the backward translation at the IS. The finding means that the EMG activity of the TA and RF, which are the frontal muscles, is predominantly enhanced by the forward translation of the support surface, but the EMG activity of the SOL, which is the back muscle, is predominantly enhanced by the backward translation of the support surface. The body leans forward after the backward translation, but leans backward after the forward translation (Nashner, 1980). Thus, it is likely that the frontal muscles (i.e., TA and RF) are activated by the backward weight shift induced by the forward translation of the support surface, but the back muscle (i.e., SOL) is activated by the forward weight shift induced by the backward translation during gait. This view is well compatible with the direction-dependent responses in stance (Nashner, 1980; Horak and Nashner, 1986). Common neural mechanism shared by the stance leg muscle responses to the perturbation during gait and the leg muscle responses to perturbation in stance is likely present.

The EMG activity of the RF after the forward translation was greater than that after the backward translation, as consistent with the finding on the TA. The EMG was tested 0–300 ​ms after the perturbation. One stride cycle of gait in humans is approximately 1000 ​ms (Blanc et al., 1999; Hausdorff et al., 1995). Stance phase of one leg shares 60% of the stride cycle (Kirtley et al., 1985; Blanc et al., 1999). Accordingly, the duration of the stance phase is approximately 600 ​ms. Thus, the change in the EMG amplitude 0–300 ms after the translation at the IS means the perturbation-induced EMG activity in the time window between the IS and MS of the gait cycle. The RF plays a role for keeping knee extension to avoid collapsing the knee due to weight loading in the time window between the IS and MS of the gait cycle (Winter and Yack, 1987, Andersson et al., 1997; Annaswamy et al., 1999; Watanabe et al., 2014). When the forward translation is given, then, the foot goes forward relative to the body above the ankle, and leans the body backward, causing greater contraction of the RF for supporting the body weight to avoid the knee collapsing. Thus, the present finding is explained by the view that the response of the RF to the forward translation is greater when the translation is given at the IS so that the RF maintains the knee extended to avoid collapsing of this joint.

The greater EMG amplitude after the forward translation may be derived from the different safety margin. Forward translation of the support surface causes backward leaning of the body. The center of pressure moves backward when the support surface moves forward (Jacobs et al., 2008). This means that the center of pressure moves to the heel when the support surface moves forward. The recovery of the body stability is difficult when humans maintain standing position over the heel. Thus, the response to the forward translation of the support surface is more difficult and fearful than the response to the backward translation of the support surface. More fearful situation in stance causes postural threat causing change in the postural control (Carpenter et al., 2001; Brown et al., 2006; Davis et al., 2009; Tanaka et al., 2013). Taken together, the direction-dependent the safety margin induced by the support surface translation, causing direction-dependent postural threat, may be the cause of the direction-dependent responses of the leg muscles to the perturbation during gait.

### 4.4 Time dependency

The EMG response was absent when the translation was given at the LS in previous studies (Nashner, 1980; Tang and Woollacott, 1999). The EMG response to the forward translation at the IS was largest among the perturbation times in previous studies (Nashner, 1980; Tang et al., 1998; Tang and Woollacott, 1999). Nevertheless, those findings were derived from the visual inspection, and were not derived from the statistical comparison among the perturbation times for the data across all participants. In the present study, statistical comparison of the across-participants mean EMG amplitude 0–300 ms after the translation was made among the translation time points. The increase in the EMG amplitude 0–300 ms after the forward translation at the IS was greater than the other translation time points in the TA and RF, that after the backward translation at the IS was greater than the other translation time points in the SOL, and the decrease in the EMG amplitude after the forward translation at the IS was greater than the other translation time points in the SOL. Thus, the present finding is the first to demonstrate statistically that the perturbation-induced change in the EMG activity is greatest at the IS among the translation time points (IS, MS, and LS).

The base of support is small and unstable at the moment at which the heel contacts the ground. It has been considered that the largest response is likely induced by the perturbation at the IS because the posture is most unstable at this phase (Tang and Woollacott, 1999). Moreover, fast response is required at the moment of the foot contact (van der Linden et al., 2007). Thus, the greatest EMG activities in the stance leg muscles other than the BF when the translation was given at the IS may be due to such an unstable situation and requirement of the rapid response at the IS.

### 4.5 Methodological considerations

On the one hand, humans cannot fully predict the upcoming perturbation, when they have not experienced it. On the other hand, when the same perturbation is repeatedly experienced several times as conducted in the present study, adaptation to the perturbation, causing the change in the response to the perturbation, may occur. However, in the present study, the experimental conditions were randomly assigned each trial, indicating that the effect of adaptation must have equally influenced across the conditions. Thus, the present findings, that the responses are dependent on the time and direction of the support surface translation, are not influenced by adaptation to the perturbation.

Another methodological concern on the present study is short walkway and the small number of steps during gait. The walkway was as short as 236 cm and the number of the steps was only five. A previous study has shown that the step length and step width are similar among first three steps of gait initiation, but the velocity and the step time in the first step are different from the second and third steps (Okada et al., 2011). Thus, the first step of the gait is likely in the acceleration phase of the gait cycle. In the present study, the earliest perturbation (translation at the IS) was given at the second step after initiating gait. Thus, even the earliest translation at the IS, the gait cycle at this time is not in the acceleration phase. Accordingly, the influence of the short walkway and small number of the steps on the present findings must be minor.

Finally, in the present study, the responses were tested only on the support leg during gait. Postural response to perturbation occurs not only in the stance leg but also in the swing leg. Thus, lack of the data on the swing leg response is one major limitation of the present study.

### 4.6 Conclusions

The EMG activity of the TA and RF after the forward translation of the support surface was greatest at the IS. In the SOL, the response to the forward translation was smallest and that to the backward translation was greatest for the translation at the IS. Those indicate that the change in the EMG activity of the stance leg muscles is greatest when the translation is given at the IS. The change in the EMG activity after the forward translation at the IS was greater than that after the backward translation in the TA and RF, indicating that the change in the EMG activity of the frontal leg muscles is dependent on the direction of the support surface translation particularly at the IS.

## Declarations

### Author contribution statement

Shiho Fukuda, Koichi Hiraoka: Conceived and designed the experiments; Performed the experiments; Analyzed and interpreted the data; Contributed reagents, materials, analysis tools or data; Wrote the paper.

Hitoshi Oda, Taku Kawasaki, Yasushi Sawaguchi, Masakazu Matsuoka, Ryo Tsujinaka: Performed the experiments.

### Funding statement

This research did not receive any specific grant from funding agencies in the public, commercial, or not-for-profit sectors.

### Data availability statement

Data will be made available on request.

### Declaration of interests statement

The authors declare no conflict of interest.

### Additional information

No additional information is available for this paper.
